# Bone Marrow Injury Induced via Oxidative Stress in Mice by Inhalation Exposure to Formaldehyde

**DOI:** 10.1371/journal.pone.0074974

**Published:** 2013-09-11

**Authors:** Yuchao Zhang, Xudong Liu, Cliona McHale, Rui Li, Luoping Zhang, Yang Wu, Xin Ye, Xu Yang, Shumao Ding

**Affiliations:** 1 Section of Environmental Biomedicine, Hubei Key Laboratory of Genetic Regulation and Integrative Biology, College of Life Science, Central China Normal University (CCNU), Wuhan, China; 2 Genes and Environment Laboratory, Division of Environmental Health Sciences, School of Public Health, University of California, Berkeley, California, United States of America; UAE University, Faculty of Medicine & Health Sciences, United Arab Emirates

## Abstract

**Objective:**

Formaldehyde, a ubiquitous environmental pollutant has been classified as a human leukemogen. However, toxicity of formaldehyde in bone marrow, the target site of leukemia induction, is still poorly understood.

**Methodology/Principal Findings:**

To investigate bone marrow toxicity (bone marrow pathology, hematotoxicity) and underlying mechanisms (oxidative stress, inflammation, apoptosis) in formaldehyde-exposed mice. Male Balb/c mice were exposed to formaldehyde (0, 0.5, and 3.0 mg/m^3^) by nose-only inhalation for 8 hours/day, over a two week period designed to simulate a factory work schedule, with an exposure-free “weekend” on days 6 and 7, and were sacrificed on the morning of day 13. Counts of white blood cells, red blood cells and lymphocytes were significantly (*p*<0.05) decreased at 0.5 mg/m^3^ (43%, 7%, and 39%, respectively) and 3.0 mg/m^3^ (52%, 27%, and 43%, respectively) formaldehyde exposure, while platelet counts were significantly increased by 109% (0.5 mg/m^3^) and 67% (3.0 mg/m^3^). Biomarkers of oxidative stress (reactive oxygen species, glutathione depletion, cytochrome P450 1A1 and glutathione s-transferase theta 1 expression), inflammation (nuclear factor kappa-B, tomour necrosis factor alpha, interleukin-1 beta), and apoptosis (activity of cysteine-aspartic acid protease 3) in bone marrow tissues were induced at one or both formaldehyde doses mentioned above.

**Conclusions/Significance:**

Exposure of mice to formaldehyde by inhalation induced bone marrow toxicity, and that oxidative stress, inflammation and the consequential apoptosis jointly constitute potential mechanisms of such induced toxicity.

## Introduction

Formaldehyde (FA) is a ubiquitous chemical that is used in a large number of industrial activities and found in many consumer products [1], [2]. Given its economic importance and widespread use, many people are exposed to FA environmentally and/or occupationally [3] and mainly by inhalation [4]. Though FA occurs naturally in all living organisms, excessive levels can induce toxicity. Acute and chronic inhaled FA have been associated with various toxic effects, including hepatotoxicity, neurotoxicity, reproductive toxicity, respiratory toxicity, and cancer, in epidemiological and animal studies [5]–[9]. The International Agency for Research on Cancer (IARC) [10] and the U.S. National Toxicology Program [11] have both classified FA as a human leukemogen based on epidemiological studies that suggest an increased risk of leukemia; the biological plausibility of FA-induced leukemia is controversial [12] however, because limited information is available on the ability of FA to disrupt hematopoietic function [13] and on mechanisms of leukemia induction.

There are limited comprehensive studies on toxicity of inhaled FA in bone marrow (BM), the site of origination of all blood cells from hematopoietic stem cell (HSC) [14] and target site for leukemia induction, have been reported. Oxidative stress is thought to underlie carcinogenesis in humans induced by chemical exposure [15], [16] and is a proposed mechanism of leukemogenesis induced by the leukemogen benzene [17]. Oxidative stress in HSC can lead to DNA damage, premature senescence, and loss of stem cell function [18]. Increased levels of oxidative stress can induce inflammation and subsequently cell apoptosis [19]. FA has been shown to induce oxidative stress in mouse brain, lung and liver following exposure by inhalation [20]. However, induction of oxidative stress, inflammation and apoptosis in BM of animal exposed to FA by inhalation have not been comprehensively measured though one study.

The aim of our study was to determine whether FA induced oxidative stress, inflammation and apoptosis in the BM of male Balb/c mice exposed to FA (0, 0.5, 3.0 mg/m^3^) by inhalation for two weeks for 8 hours/day, for 5 consecutive days per week, to mimic occupational exposure. Exposure levels were based on the current and historic Chinese occupational standards. We used histological, biochemical, and immunological assays to evaluate these adverse effects on BM after FA inhalation, and to investigate potential underlying mechanisms.

## Materials and Methods

All experimental procedures were approved by the Office of Scientific Research Management of Central China Normal University (Wuhan, China) with a certification of Application for the Use of Animals dated 8 November 2011 (approval ID: CCNU-SKY-2011-008).

### Reagents and kits

Formalin solution (10%), 2′,7,- dichlordihydrofluorescein (DCFH-DA), 3-Carboxy-4-nitrophenyl disulfide (DTNB) were obtained from Sigma-Aldrich (St Louis, MO, USA); Trizol reagent was purchased from Invitrogen (Carlsbad, CA, USA); M-MLV, OligodT and DNA maker were obtained from TAKARA (Otsu, Shiga-ken, Japan); 2×Taq Master Mix, RNase-Free Water were obtained from CWBIO (Beijing, China). All other chemicals were of analytical grade.

Mouse EILSA kit for NF-κB was purchased from Blue Gene (Shanghai, China). Mouse EILSA kits for TNF-α and IL-1β were purchased from eBioscience (San Diego, CA, USA). Mouse caspase-3 activity assay kit was purchased from Beyotime (Nanjing, Jiangsu, China). If not otherwise stated, all chemicals and reagents were used without further purification.

### Animals

Specific-pathogen-free male Balb/c mice (5–6 weeks old, 22±1.5 g) were purchased from the Hubei Province Experimental Animal Center (Wuhan, China) and housed in standard environmental conditions (12 h light-dark cycle, 50–70% humidity and 20–25°C). Food and water were provided *ad libitum*. The mice were quarantined for at least 7 days before study initiation. Nine mice were included in each group to provide sufficient statistical power.

### Preparation of gaseous FA

Gaseous FA was prepared from 10% formalin. FA vapor was generated and all environmental parameters were monitored with HOPE-MED 8052 inhalation equipment (Hope-med Company, Tianjin, China). Exposure levels were based on the current (0.5 mg/m^3^, 2002, 2007) and historic (3.0 mg/m^3^, 1979) Occupational Exposure Limits established by the Chinese Ministry of Health [21]. Air temperature, relative humidity and airflow rate were maintained at (22±2)°C, (50±5)% and (1.65±0.15) m^3^/h, respectively. During exposure, FA concentrations were monitored every 2 hour using a Gaseous FA Analyzer (4160-2, Interscan, Simi Valley, CA, USA).

### Experimental protocol

**Figure 1:**
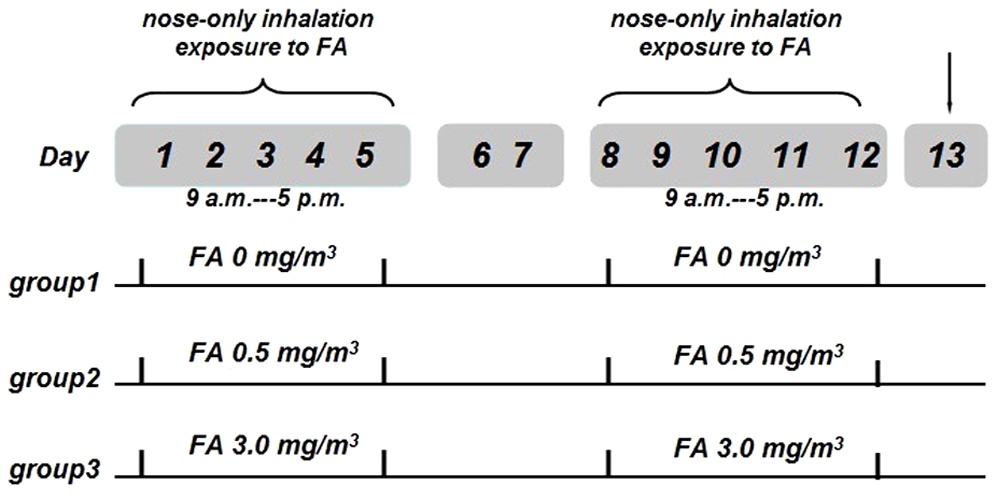
Study protocol. group 1 (n = 9): control group, exposed to 0 mg/m^3^ FA; group 2 (n = 9): exposed to 0.5 mg/m^3^ FA; group 3 (n = 9): exposed to 3.0 mg/m^3^ FA.

Mice were divided randomly into three experimental groups of 9 animals. To simulate the major exposure route for occupational workers in China (8 hours/day, 5 working days per week), mice were exposed to FA (0, 0.5, and 3.0 mg/m^3^) via nose only inhalation from 9 a.m. to 5 p.m., 8 hours a day, 5 consecutive days in a week, and totally lasted for two weeks. The detailed protocol of this study is shown in **Figure 1**.

### Sample preparation

At 9 a.m. on the 13th day, blood samples (30 µL) were collected from mice caudal vein. The mice were then sacrificed by cervical dislocation and femurs were collected for subsequent assays.

### Complete blood count (CBC)

After FA exposure, CBC was conducted on a Blood Cell Analyzer (MTN-21, Motenu, Changchun, Jilin,China) according to the manufacturer's instructions. The number of leucocyte (WBC), erythrocyte (RBC), lymphocytes (LYM), neutrophilic granulocytes (GRA), intermediate cells (MON), and platelets (PLT) were counted.

### Histological assay

Histopathology slice preparation from the femurs was obtained from 2 animals in each group. Samples were incubated in the fixing solution (saturated 2, 4, 6-trinitrophenol: formalin: glacial acetic acid [15∶5∶1 v/v/v]) at room temperature for 24 h and then stained with hematoxylin and eosin (H&E) [22]. Finally, the tissues were embedded in paraffin, following by sectioning into slices with a sickness of 10 µm, and then examined under a microscope (Leica DM 4000B, Berlin, Germany). Tissue sections were examined qualitatively by two experienced pathologists in a blinded fashion.

### Determination of reactive oxygen species (ROS)

ROS levels in the mouse BM were determined by DCFH-DA fluorescent assay [23] with minor modifications. After exposure, femurs were collected and their ends were cut using ophthalmic scissors. The marrow was rinsed with 1 mL pre-warmed 1×PBS (pH = 7.4) five times to collect the marrow suspension, following by a centrifugation at 100 g for 3 min to collect the precipitated bone marrow cells. Cells were counted using a hemacytometer and adjusted to a density of 10^7^/mL using 1×PBS. DCFH-DA was then added into 300 µL cell suspension (10^6^/mL) to form a final concentration of 10 µmol/L and incubated at 37°C for 30 min. After incubation, cells were washed with 1×PBS and re-suspended in 300 µL 1×PBS. The cell suspension (200 µL) was transferred into a 96-well microplate and the fluorescence intensity at 525 nm was measured under 488 nm excitation by a fluorescence reader (FLx 800, BioTek Instruments, Vinooski, VT, USA).

### Glutathione (GSH) depletion assay

The GSH levels in mouse BM were determined by the DTNB fluorescent assay [24] with minor modifications. In this assay, GSH reacts with DTNB in the dark and forms yellow compounds. First, 50 µL of 10% trichloroacetic acid (TCA) was added into 200 µL cell suspension (10^6^/mL) and mixed well. Samples were then centrifuged at 9,300 g for 5 min and supernatants were collected into a new vial. After pH adjustment to 7.5 with 2 mol/L NaOH to optimize the condition for the color development reaction with DTNB, 50 µL reactant was transferred into the microplate with addition of 150 µL of DTNB (60 µg/mL, diluted 50-fold from DMSO-dissolved stock solution). Samples were incubated in dark at room temperature for 5 min, prior to an analysis of both experimental and standard samples (reductive GSH, Sigma-Aldrich, St Louis, MO, USA) using a microplate reader at 412 nm. Based on the standard curve, the concentrations of GSH (nmol/L) in each sample were calculated as OD_412_/0.0023 (R^2^ = 0.997). The protein concentration of the sample was determined using Lowry assay [25].

### Expression of cytochrome P450 1A1 (CYP1A1) and glutathione s-transferase theta 1 (GSTT1) mRNA

CYP1A1 and GSTT1 are representative of phase I and phase II metabolic enzymes and they are important in maintaining reduction-oxidation balance. Expressions of CYP1A1 and GSTT1 mRNA in the BM were detected by reverse transcription and polymerase chain reaction (RT-PCR). Total RNA was extracted from each femur by Trizol and cDNA was generated by reverse-transcription using oligodT primers. PCR was carried out in a DNA thermal cycler (C1000, Bio-Rad, San Diego, CA, USA) at 94°C for 3 min, followed by 30 cycles of 94°C for 1 min, 56°C (CYP1A1) or 60°C (GSTT1) for 1 min, and 72°C for 1 min. The following PCR primers (GenScript, Nanjing, Jiangsu, China) were shown in **Table 1**. β-actin was used as the internal control.

**Table 1:** Primers used for RT-PCR.

Target	Primer	Primer sequence
CYP1A1	Forward	5′- AGTATTTGGTCGTGTCAGTAG -3′
	Reverse	5′- TCCAGGGAAGAGTTAGGC -3′
GSTT1	Forward	5′- TCAGCGATGCGTTTGCC -3′
	Reverse	5′- GCCAGTGTTTCAGGAGGTA -3′
NF-κB	Forward	5′- CCATCGGTGGGATTACATTC -3′
	Reverse	5′- CTCCTCGTCATCACTCTTGG -3′
β-actin	Forward	5′- GCTGTCCCTGTATGCCTCT -3′
	Reverse	5′- GGTCTTTACGGATGTCAACG -3′

Amplification products were separated by 1.5% agarose gel electrophoresis and visualized by ethidium bromide staining. Images were captured and the levels of expression of CYP1A1 and GSTT1 relative to β-actin were quantified with Bio-imaging System (Gel Doc XR+, Bio-Rad, San Diego, CA, USA).

### Expression of nuclear factor kappa-B (NF-κB) mRNA and protein

Expressions of NF-κB mRNA in BM were detected by RT-PCR using primers as shown in **Table 1**. RT-PCR was conducted as for CYP1A1 and GSTT1, with an annealing temperature of 56°C. NF-κB protein levels were measured by a commercial ELISA kits with a sensitivity of 0.1 ng/mL.

### Cytokine levels of tumour necrosis factor alpha (TNF-α) and interleukin-1 beta (IL-1β)

Levels of TNF-α and IL-1β were measured using commercial ELISA kits, according to the manufacturer's instructions. The sensitivities of the ELISA kits were 8 pg/mL for TNF-α and 80 pg/mL for IL-1β.

### Activity of cysteine-aspartic acid protease 3 (Caspase-3)

Caspase-3 plays a pivotal role in apoptosis. The activation level of caspase-3 was determined with the mouse caspase-3 activity assay kit according to the manufacturer's instructions. The assay is based on spectrophotometric detection of the chromophore *p*-nitroanilide (*p*NA) after its cleavage from the labeled substrate DEVD-*p*NA. Absorbance of the chromophore *p*NA produced was measured at 405 nm.

### Statistical analysis

Analysis of variance (ANOVA) was applied on statistical analysis. The data was presented as the mean ± standard error of the mean. Statistical graphs were generated using Origin 8.0 software (OriginLab, Berkeley, CA, USA). One-way ANOVA combined with Fisher's protected t-test was used to determine the significance of differences between groups. *p*<0.05 was considered as significant difference and *p*<0.01 was considered as extremely significant difference. Data analyses were carried out using SPSS ver13 (SPSS, Chicago, IL, USA). The measurements of all the data are carried out more than three times repeating.

## Results

### Effect of FA on complete blood count

**Figure 2:**
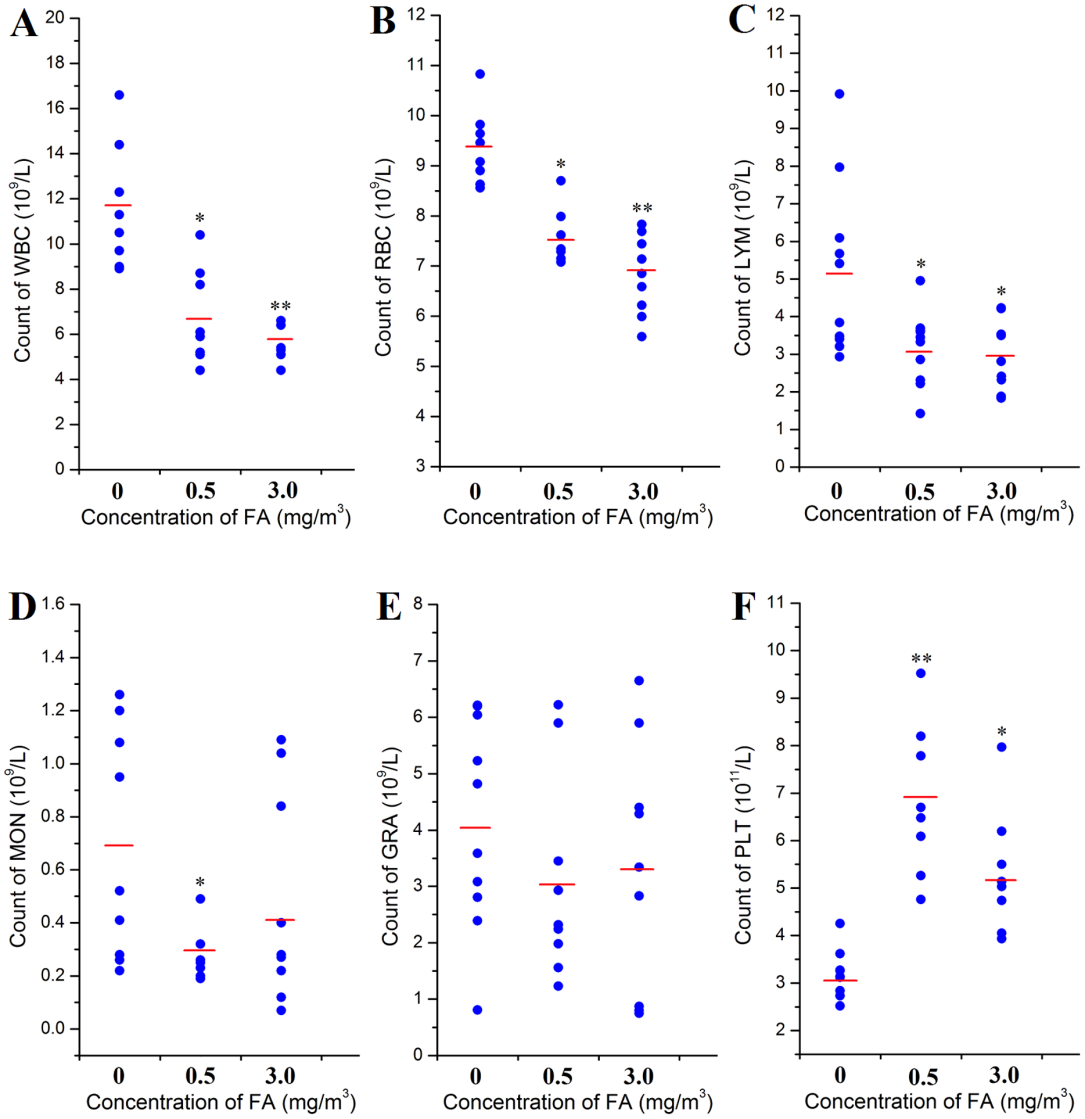
Effect of FA on complete blood count. Complete blood cell counts (for each mouse in each group and group mean) following 2-week exposure to FA (0.5 and 3.0 mg/m^3^) are presented. A: WBC (×10^9^/L); B: RBC (×10^9^/L); C: LYM (×10^9^/L); D: MON (×10^9^/L); E: GRA (×10^9^/L); F: PLT (×10^11^/L). ANOVA was used to measure the difference in group mean cell counts, *: *p*<0.05 and **: *p*<0.01, compared with control group (0 mg/m^3^ FA). All results were obtained from at least 3 paralleled samples.

After FA exposure, counts of WBC, RBC, LYM and PLT were significantly changed relative to unexposed control mice (**Figure 2**). WBC counts were significantly decreased by 43% (0.5 mg/m^3^; *p*<0.05) and 52% (3.0 mg/m^3^; *p*<0.01), respectively (Figure 2A). RBC counts were decreased by 17% (0.5 mg/m^3^; *p*<0.05) and 27% (3.01mg/m^3^; *p*<0.01), respectively (Figure 2B). Counts of LYM were significantly decreased by 39% and 43% (0.5 and 3.0 mg/m^3^, respectively; *p*<0.05; Figure 2C). However, PLT counts showed the opposite response, they were significantly increased by 109% (0.5 mg/m^3^; *p*<0.01) and 67% (3.0 mg/m^3^; *p*<0.05), respectively (Figure 2F). The counts of MON and GRA in exposed groups were not significantly altered relative to controls.

### Bone Marrow Histology

**Figure 3:**
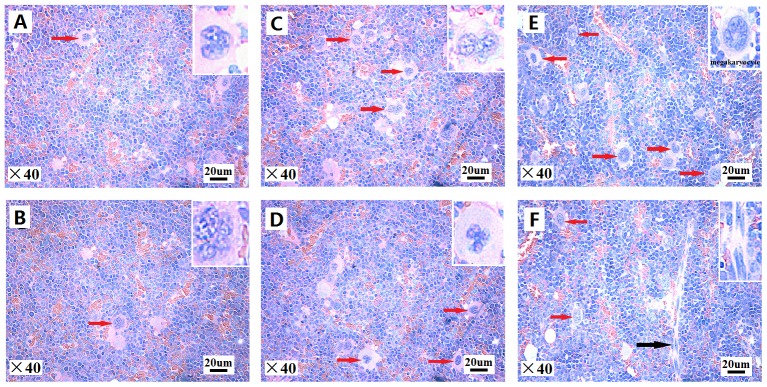
Effects of FA on bone marrow histology. Bone marrow from mouse femurs following FA exposure were subjected to hematoxylin and eosin staining and visualized at ×40 magnification. Representative images from two unexposed control mice (0 mg/m^3^ FA) (A) and (B), two mice exposed to 0.5 mg/m^3^ FA (C) and (D), and two mice exposed to 3.0 mg/m^3^ FA (E) and (F), are shown. MKs are indicated by red arrows and myelofibrosis is indicated by a black arrow.

Mouse BM histology was examined in femurs from control and exposed mice after FA exposure (**Figure 3**). BMs from unexposed control mice (0 mg/m^3^) showed normal cell morphology. Figures 3A and B. Abnormalities were detected in the marrows from exposed mice; the number of megakaryocytes (MKs) exhibited a positive correlation with the FA dose (0.5 and 3.0 mg/m^3^), shown in Figures 3C, D, E, and F and the myelofibrosis was observed at the 3.0 mg/m^3^ dose, shown in Figure 3F.

### Effect of FA on oxidative stress

**Figure 4:**
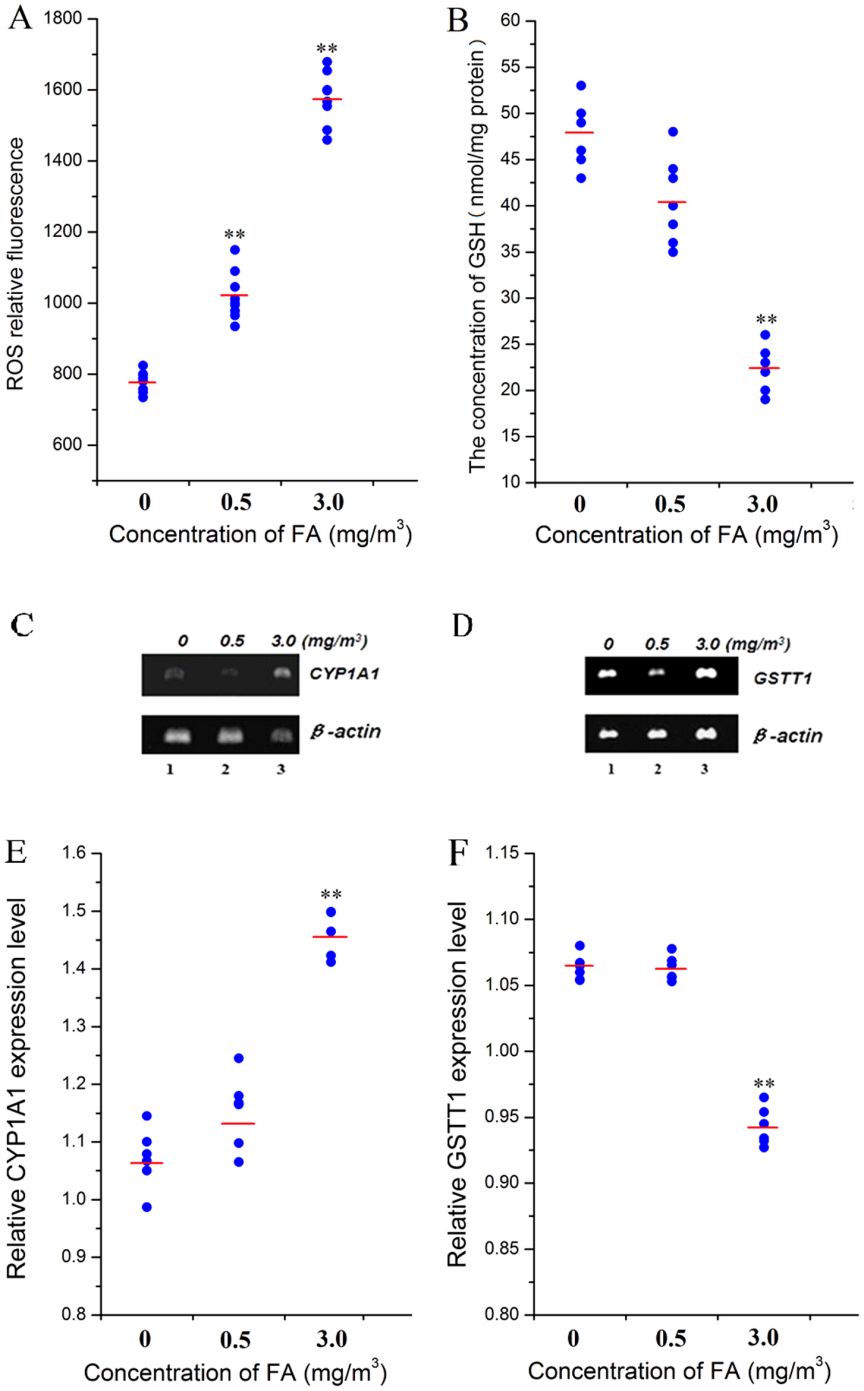
Effect of FA on oxidative stress in bone marrow. Markers of oxidative stress were measured in the bone marrow of FA exposed and control mice. Individual marker levels for each mouse and group means are shown for each dose group. (A) ROS level (relative florescence); (B) GSH (nmol/mg protein); (C) Agarose gel image of CYP1A1 mRNA expression (β-actin as internal control); (D): Agarose gel image of GSTT1 mRNA expression (β-actin as internal control); (E) Relative CYP1A1 expression level; (F): Relative GSTT1 expression level. **: *p*<0.01, compared with control group (0 mg/m^3^ FA).

The effects of exposure to FA by nose-only inhalation on markers of oxidative stress in bone marrow were shown in **Figure 4**. ROS levels were significantly (*p*<0.01) increased at 0.5 mg/m^3^ FA by 31% and at 3.0 mg/m^3^ FA by 102%, compared to unexposed controls, respectively as shown in Figure 4A. GSH levels were not significantly changed at 0.5 mg/m^3^ FA but significantly decreased at 3.0 mg/m^3^ FA by 54%, compared to unexposed controls (*p*<0.01, Figure 4B).

CYP1A1 mRNA expression was increased in both 0.5 (by 8%) and 3.0 mg/m^3^ (by 37%) FA compared with the control group (Figure 4C) and the increase was statistically significant (*p*<0.01) at 3.0 mg/m^3^ group (Figure 4E). GSTT1 mRNA expression was unchanged at 0.5 mg/m^3^ FA compared with untreated controls but was decreased by 13% at 3.0 mg/m^3^ (Figure 4D) and was statistically significant (*p*<0.01, Figure 4F).

### Effect of FA on NF-κB and inflammatory cytokines

**Figure 5:**
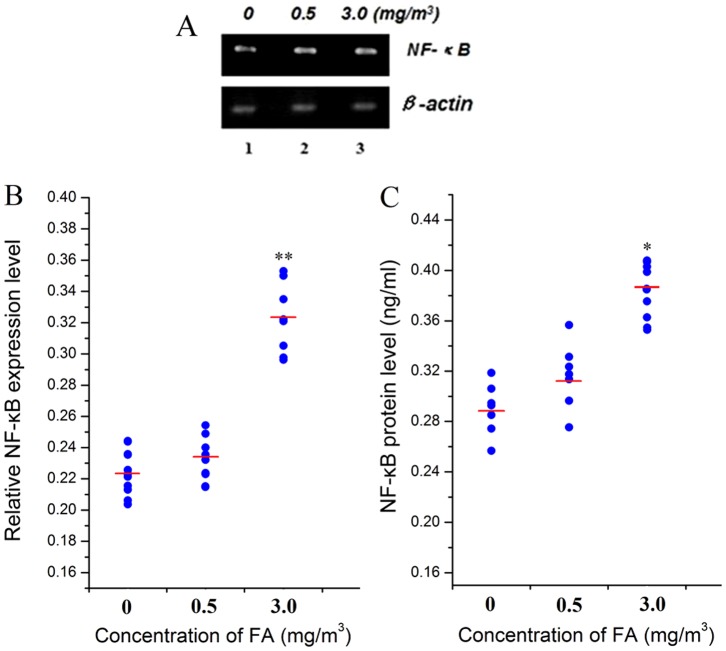
Effect of FA on NF-κB mRNA and protein levels in bone marrow. NF-κB mRNA and protein levels were measured in the bone marrow of FA exposed and control mice, by RT-PCR and ELISA, respectively. (A): Agarose gel image of NF-κB mRNA expression (β-actin as internal control); (B): Relative NF-κB expression level; (C) NF-κB protein level (ng/mL). *: *p*<0.05, compared with control group (0 mg/m^3^ FA), **: *p*<0.01, compared with control group (0 mg/m^3^ FA).

**Figure 6:**
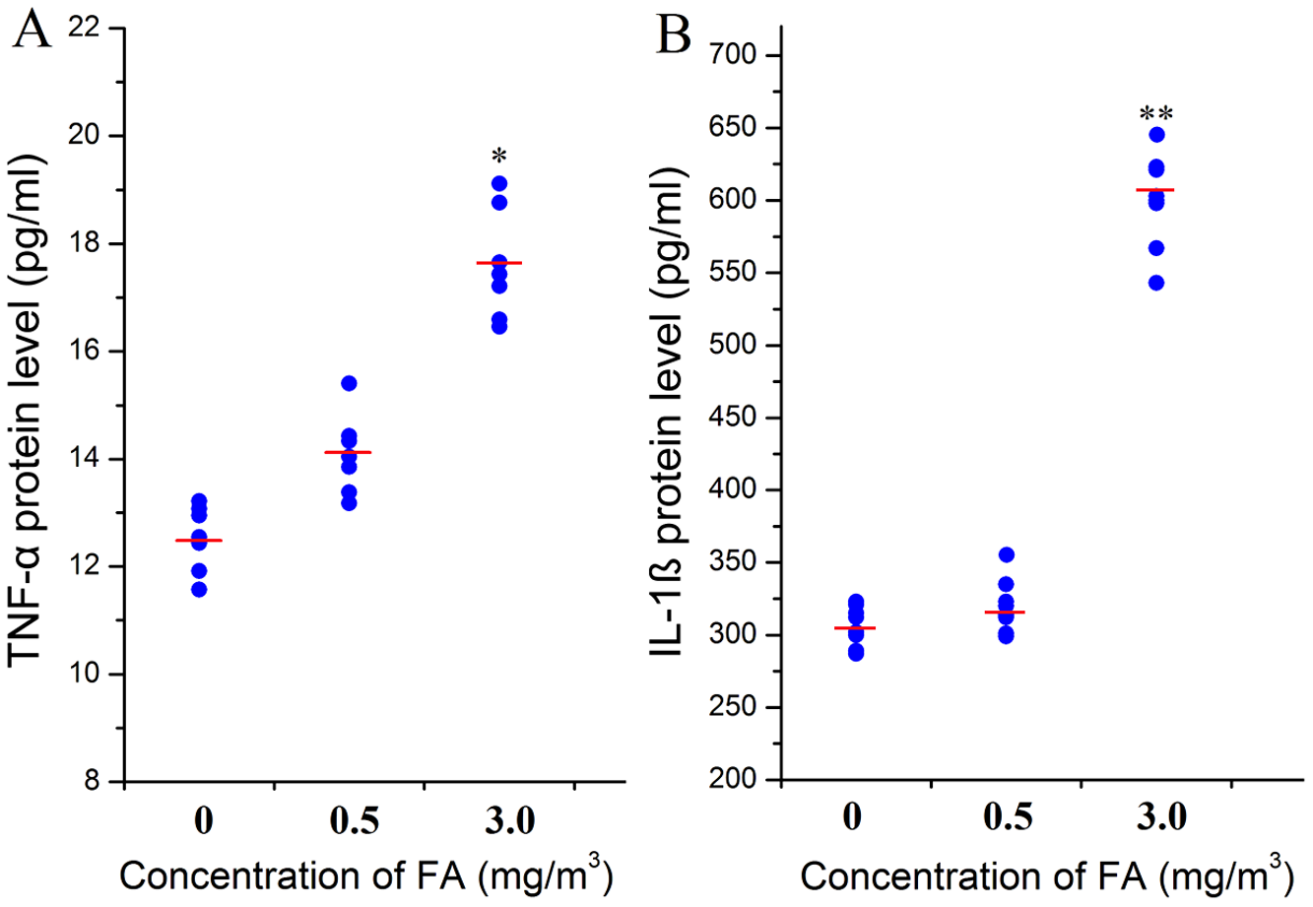
Effect of FA on inflammatory cytokines in bone marrow. TNF-α and IL-1β cytokine levels were measured in the bone marrow of FA exposed and control mice by ELISA. (A): TNF-α protein level (pg/mL). (B) IL-1β protein level (pg/mL). *: *p*<0.05, compared with control group (0 mg/m^3^ FA), **: *p*<0.01, compared with the control group (0 mg/m^3^ FA).

NF-κB mRNA expression showed an upward trend (Figure 5A) and NF-κB was significantly increased at 3.0 mg/m^3^ FA exposure group at both mRNA level (by 44%, Figure 5B,) and protein level (by 34%, Figure 5C). TNF-α levels in BM were significantly increased by 42% at 3.0 mg/m^3^ FA compared to those in the control group (*p*<0.05, Figure 6A). IL-1β levels were significantly increased by 98% at 3.0 mg/m^3^ FA (*p*<0.01, Figure 6B). These results were not significantly altered relative to controls at 0.5 mg/m^3^ FA.

### Effect of FA on apoptosis

**Figure 7:**
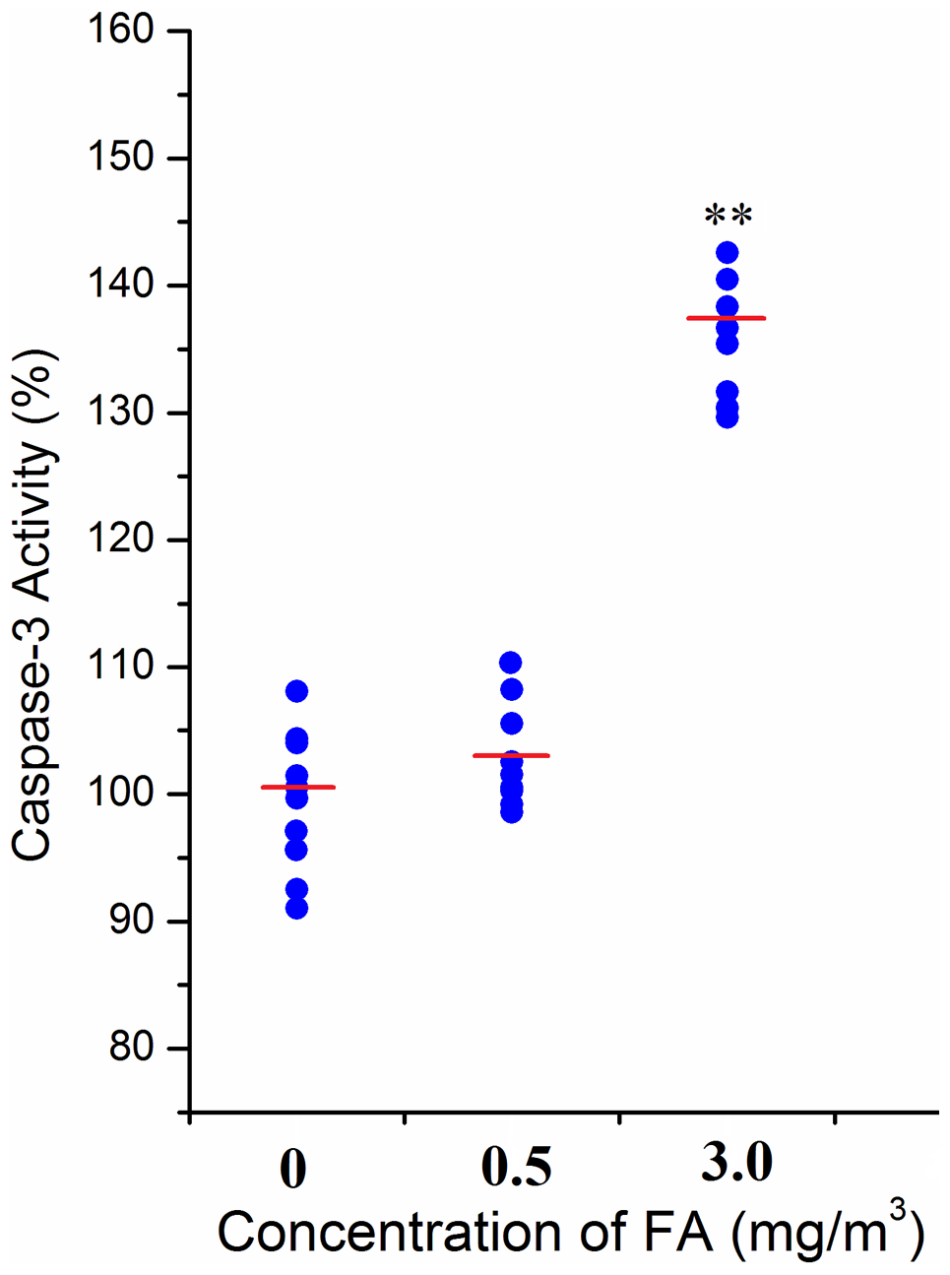
Effect of FA on apoptosis in bone marrow. Caspase-3 activity was measured in the bone marrow of FA-exposed and unexposed control mice. **: *p*<0.01, compared with control group (0 mg/m^3^ FA).

After FA inhalation exposure, caspase-3 activity was similar with control at 0.5 mg/m^3^ and significantly increased by 38% at 3.0 mg/m^3^ (*p*<0.01) as shown in **Figure 7**.

## Discussion

**Figure 8:**
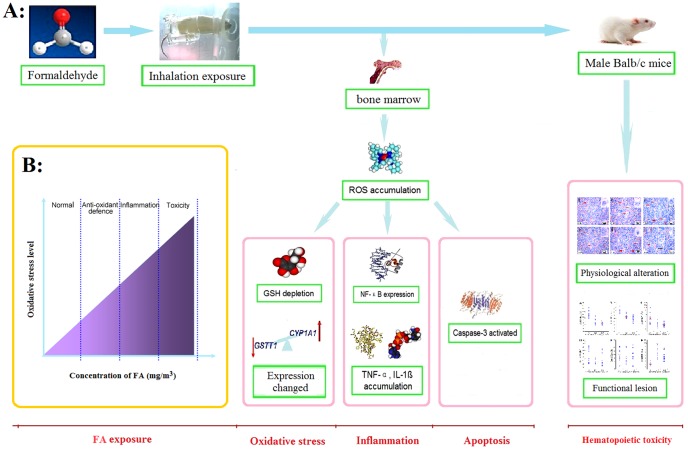
Potential mechanism of FA induced bone marrow toxicity. (A): FA exposure induced adverse effects on mice BM through generation ROS and oxidative stress-induced inflammation and apoptosis in BM. (B): At low FA concentration, the damages were slightly. With the exposure dose increased, inflammation and apoptosis occurred in BM and damages were more serious.

Although FA has been classified as a human leukemogen by IARC and U.S. National Toxicology Program, the biological plausibility of FA-induced leukemia is still controversial. Hematotoxicity data and the significant induction of leukemia-specific chromosome changes in myeloid blood progenitor cells from exposed workers in a molecular epidemiological study of FA toxicity in workers in China, were indicative of toxic effects on the BM [13]. Animal studies are amenable to the direct examination of BM toxicity. Up to date, however, there is no comprehensive studies on FA toxicity in BM, the site of leukemia induction, and its examination anomaly is one of the most important characteristics of hematopoietic system diseases [26], in exposed animals have been published. In this study, we have assessed various indices related to the BM toxicity in mice exposed to FA by nose-only inhalation and provide evidence of a potential mechanism we proposed involving oxidative stress, inflammation and apoptosis, based on the induction of multiple markers (**Figure 8**).

Hematopoiesis is a tightly regulated process resulting in the production of blood cells and its disruption can lead to altered cell distribution and disease [27]. All blood cell lineages are produced in a hierarchical manner from HSC in BM and leukemia originates from damage induced in HSC [28]. One of the clinical consequences of damage to hematopoietic stem or progenitor cells is an alternation in circulating RBC, WBC and PLT counts [13]. Currently, CBC is the most frequently ordered laboratory test in the clinical milieu and a simple CBC can rule out, confirm, or direct the attention to anemia, cancer or toxin exposure [29]. Therefore, changes in the numbers of circulating blood cells in several lineages may reflect toxicity to BM HSC. In our study, significantly altered counts of WBC, RBC, LYM (decreased) and PLT (increased) in FA-exposed mice, the results are consistent with the clinical symptoms of some hematopoietic diseases such as aplastic anaemia (AA), myelofibrosis and megakaryocytic leukemia, indirectly suggesting that FA induces BM toxicity.

Direct evidence of BM toxicity came from histological examination of the BMs of exposed and control mice, which revealed abnormalities induced by FA, including an increase in the number of MKs and myelofibrosis. Histological changes occur in hematopoietic diseases and some changes can indicate the risk developing disease [30], [31]. MKs give rise to circulating platelets (thrombocytes) and there is a relationship between the increase of MKs and PLT [32], [33], the latter which were increased in number by FA exposure in our study. However, abnormal proliferation of MKs can cause malignant diseases like megakaryocytic leukemia, characteristics of which include proliferation of abnormal MKs and myelofibrosis [34]. Myelofibrosis is also a blood cancer characterized by fibrotic BM, excessive MKs and altered hematopoiesis. Its ability to transform into leukemia increases the morbidity [35].

Together, the CBC and histology data suggest that FA induces BM toxicity, potentially increasing the risk of hematopoietic disease. The underlying mechanisms are not known. As oxidative stress is be involved in the pathophysiology of some hematopoietic system diseases [36], [37], we hypothesized that it would play a role in FA-induced BM toxicity.

The redox state of cells, the balance of oxidants, anti-oxidants and free radicals, plays an important role in cellular signaling, control of vascular tones, cell generation, and defense against microorganisms [38]. ROS, including singlet oxygen, superoxide anion, hydrogen peroxide and hydroxyl radical, are induced by cellular aerobic metabolism, inflammation, or exposure to several chemical and physical agents, such as FA [39]. *In vivo*, there are a lot of anti-oxidants to delete ROS, the imbalance between ROS and antioxidant defenses has been described as oxidative stress [40]. Oxidative stress can induce protective or adverse cellular responses in a dose-dependent manner. CYP1A1 is a phase I metabolic enzyme that plays an important role in the production of ROS during the metabolism of numerous exogenous and endogenous compounds [41], [42]. Lower oxidative stress level induces cytoprotective responses, phase II drug metabolizing enzymes and antioxidant, such as GSTT1 and GSH, are involved in the elimination of ROS [43], [44]. If the protection fails, the oxidative stress will first lead to the activation of mitogen-activated protein kinases and NF-κB cascades then induce pro-inflammatory responses. If oxidative level continue rising, the response will be overtaken by cytotoxicity, resulting in apoptosis or necrosis [44].

Several markers of oxidative stress and oxidative stress response were altered in the present study in mouse BM by FA exposure. ROS and CYP1A1 levels were were significantly increased, suggestive of the induction of oxidative stress, and GSH and GSTT1 were significantly decreased, suggesting the overwhelming of defense meachanisms. Moreover, CYP1A1 and GSTT1 are essential in the carcinogen activation and detoxification. GSTT1 polymorphisms were shown to modulate the genotoxic effects of FA exposure in one study but not in two additional studies were negative [45], [46]. Genetic variability underlying differences in balance between phase I and phase II biotransformation among individuals may influence susceptibility to environmental toxins [47].

NF-κB, a redox sensitive transcription factor, can convert oxidative stress signals into changes in the expression of genes associated with diverse cellular activities. The activation of signaling pathways via NF-κB initiates the production of pro-inflammatory mediators such as TNF-α and IL-1β [48]. As for our data, after the FA exposure, the expression of NF-κB in BM increased, which subsequently enhanced the release of TNF-α and IL-1β. These results confirmed each other and revealed that FA exposure could stimulate inflammation in BM. It has been proposed that inflammation promotes cancer development and progression, including some hematologic malignancies [49]. As a naturally occurrence, cytokine of inflammatory and immune responses, TNF-α has been involved in the pathogenesis of hematologic malignancies, such as multiple myeloma [50], [51], myelodysplastic syndrome (MDS) [52], and acute myelogenous leukemia (AML) [53], [54]. Moreover, IL-1β has been found to be produced at high concentrations by AML marrow cells and to stimulate AML growth [55]. Therefore, we suggest that, after FA exposure, the stimulated inflammation in BM might increase the risk of hematopoietic system diseases.

Based on our data, oxidative stress and inflammation were both demonstrated in BM after FA exposure, while the enhanced ROS formation can ultimately induce cytotoxicity. Various pathologies can result from the oxidative stress-induced apoptosis [56]. Caspases are a family of cysteine proteases, which have proteolytic active to cleave proteins at aspartic acid residues. Once caspases are initially activated, there seems to be an irreversible commitment towards apoptosis [57]. Caspase-3 activity plays vital role in the caspase-dependent mechanism [58]. The activation of caspase-3 indicated that apoptosis occurred in mice BM after FA exposure. Apoptosis of BM cells has been related to some hematopoietic system diseases, e.g. AA [59] and MDS [60]. The results implied that after FA exposure, the mice were more vulnerable to hematopoietic diseases.

In conclusion, we have demonstrated that inhalation exposure of mice to FA, at levels and duration similar to human occupational exposure, induces BM toxicity, and that oxidative stress, inflammation and apoptosis may be underlying mechanisms. All results that collectively suggested inhalation exposure to FA could induce the adverse effects on BM in mice, which might increase the risk of hematopoietic system diseases. Further study is needed on validating in other mice models and additional markers of oxidative, inflammation, and apoptosis. Considering the multiple ways in which human exposure to FA, inhibition oxidative stress after FA exposure is urgently needed.
